# Syphilitic Ulceration of the Eye-Lid (Conjunctiva) in the Infant

**Published:** 1882-06-20

**Authors:** A. W. Calhoun

**Affiliations:** Atlanta; Professor of Eye, Ear and Throat Diseases in the Atlanta Medical College


### SYPHILITIC ULCERATION OF THE EYE-LID (CONJUNCTIVA) IN THE INFANT.

BY A. W. CALHOUN, M. D.,

**Professor of Eye, Ear and Throat Diseases in the Atlanta Medical College.**

In the adult, we are accustomed to seeing syphilis in some form or other, take hold of almost any and every part of the body; and even in the infant, it is not unusual that the disease manifests itself secondarily in various ways; but it is so seldom the case that syphilitic ulceration takes place upon the mucous membrane of the eye-lid, that I am induced to report the following history:

It is now eight years since Mr. A. J. M. contracted primary syphilis. The disease progressed uninterruptedly till a portion of the upper jaw and a part of the bones of the nose were destroyed, without, however, much disfigurement. Upon the supposition that he had been cured, he married four years ago, and in due course of time a son was born, who was, at birth, and up to the present remains, a specimen of robust health.

One year ago another son was born, who, from birth, has been a typical specimen of hereditary syphilis.* Copper colored blotches first appeared over the entire body, then eczema upon the scalp with enlargement of the cervical glands, then ulceration of the right pre-auricular glands. Two months back, phlyctenular corneitis began, the phlyctenules running together and breaking down into deep corneal ulcers in each eye, causing the pain and photophobia and other distressful symptoms so often met with in children with so-called "scrofulous sore-eyes."

The eye disease had been in existence two months when the child came under my observation, and in addition to the extensive ulceration of each cornea, the following condition of the lid appeared: The left upper lid was swollen and angry-looking, and extended downwards over and covered the lower lid. Upon everting the lid, a very characteristic syphilitic ulcer was found to occupy the centre of the conjunctiva, being near the size of a three cent silver coin. It had the ragged and undermined edges, the dirty surface and other marked appearances, which so readily distinguishes the specific ulcer from all other varieties. Aside from the father's history and the general condition of the child, the ulcer itself left no doubt as to the diagnosis.

The little patient was immediately put upon anti-syphilitic treatment. Iodide potass. was given in 2 grain doses and gradually pushed to 15 grains 3 times daily, with the happiest result. The sulphide of calcium was also given in 1/6 grain doses, mixed with a little sugar of milk, for its alterative effect. As local applications, the sulph. atropia (gr. j to ℥j) was used upon the eyes for its curative influence upon the corneal ulcers, and an ointment of the yellow oxide of mercury (gr. 1/2 to ʒj) applied to the edges of the lids which had become excoriated by the profuse lachrymation. The eyes were kept as free from matter as practicable, but throughout the treatment, no application was made to the conjunctival ulcer. The child has now been under treatment one month, and the syphilitic ulceration of the lid and every external symptom of the disease has disappeared, though the phlyctenular inflammation of the cornea still remains to a slight degree.

The history of this case is recorded, not because of the rapid recovery under this particular treatment, but because of its unfre-

*NOTE.—No attempt will be made here to explain why the first child was free from syphilis, and the second had it. The mere fact is given.

quent occurrence. In so far the case is of special interest. I have met with a few similar cases amongst adult hospital patients, but neither in a very large hospital experience, nor in a private practice of a good number of years and comprising many thousands of patients, have I ever before seen the disease upon the mucous membrane of the eye-lid, in the infant.

